# Supplementary material for: Need for affect, need for cognition, and the desire for independence
Source: PLoS One. 2023 Feb 9;18(2):e0280457. doi: 10.1371/journal.pone.0280457 (PMC9910755; doi:10.1371/journal.pone.0280457)
Supplement: S1 Appendix — (PDF) [file pone.0280457.s001.pdf]

Online Appendix for:
“Need for Affect, Need for Cognition, and the Desire for Independence”

Contents

A Weighting procedures **2**

B Comparison of sample survey to the population statistics **3**

C Descriptive statistics **4**

D Need for Affect Scale **5**

D.1 Item wordings 5

D.2 Correlation matrix 7

D.3 Factor loadings 7

E Need for Cognition Scale **8**

E.1 Item wordings 8

E.2 Correlation matrix 11

E.3 Factor Loadings 13

F Personality traits measures **14**

F.1 Item wordings 14

F.2 Correlations across items 16

G Correlations across all variables and measures **17**

H Full regression models **18**

A Weighting procedures

For weighting purposes, I define $S = 1$ as my target sample (from the BOP) and $S = 0$ as my convenience sample (Internet survey). With both samples merged, I define my weights by estimating a standard logistic regression to obtain fitted values of S as:

$$\hat{S}(x) = E[S|X] = (\frac{\mathrm{e}^{a+\mathbf{X}}}{1+\mathrm{e}^{a+\mathbf{X}}}), \tag{1}$$

where $\hat{S}(x)$ is the predicted probability that a given observation belongs to the target sample instead of my convenience sample and $\mathbf{X}$ is the vector of covariates that are matched between the samples. For estimation based on the representative sample, I define weights as follows:

$$\omega(S,x) = (1-S)\frac{\hat{S}(x)}{1-\hat{S}(x)}. \tag{2}$$

By this definition, when $S = 1$ (i.e., observation that belongs to the target sample) equation eq:2 becomes $\omega(S,x) = 0$, and so excluded from the analysis. When $S = 0$ (i.e., observation that belongs to my convenience sample) then equation eq:2 becomes $\frac{\hat{S}(x)}{1-\hat{S}(x)}$. To sum up, if I denote $\hat{S}(x)$ simply as P, the weight for an observation in my convenience sample is $\frac{P}{1-P}$ in my estimations.

P is transformed to have an average of 1 to maintain the original sample size.

B Comparison of sample survey to the population statistics

Table B.1: Comparison of the Descriptive Statistics of the Internet Survey and the BOP (probabilistic survey)

	BOP (probabilistic survey)			Internet survey (opt-in)			Two-sample differences		
Variables	N	Mean	SD	N	Mean	SD	Diff. in means	t-value	p-value
**Age**	2,249	49.23	17.26	37,055	48.61	15.12	.62	1.88	.06
**Political ideology**	1,971	2.028	1.224	37,055	2.005	1.140	.02	.83	.40
**Gender, female**	2,249	.509	.500	37,055	.469	.499	4.06	3.74	.00
**Turnout in regional elections 2012**	2,249	.831	.374	37,055	.846	.361	1.46	-1.85	.06
**Support for the Catalan secession**	2,249	.585	.493	37,055	.618	.486	3.3	-3.12	.00
**Province of residence**									
Girona	2,249	.204	.403	37,055	.186	.389	1.8	2.12	.03
Tarragona	2,249	.198	.399	37,055	.202	.402	0.45	-.51	.60
Lleida	2,249	.194	.396	37,055	.201	.401	0.65	-.74	.45
Barcelona	2,249	.411	.492	37,055	.404	.491	0.7	-.65	.51
**Educational Attainment**									
College and above	2,249	.213	.409	37,055	.240	.427	2.69	-2.90	.00
HS or Some College	2,249	.467	.50	37,055	.494	.50	2.7	-2.48	.01
Lower than HS	2,249	.32	.466	37,055	.266	.442	5.39	5.60	.00
**Subjective Social Class**									
Upper/Middle - Upper	2,249	.077	.268	37,055	.083	.276	0.55	-.91	.35
Middle	2,249	.509	.500	37,055	.541	.498	3.19	-2.94	.00
Lower/ Lower-Middle	2,249	.414	.493	37,055	.376	.484	3.74	3.55	.00
**Origin**									
Immigrant	2,249	.205	.404	37,055	.172	.378	3.27	3.97	.00
2n Generation	2,249	.160	.367	37,055	.166	.372	0.52	-.64	.52
2n Generation mixed	2,249	.146	.353	37,055	.161	.368	1.58	-1.98	.05
Native	2,249	.489	.500	37,055	.501	.500	1.18	-1.08	.28
**Mother Language**									
Spanish	2,249	.550	.498	37,055	.563	.496	1.33	-1.23	.21
Both	2,249	.383	.486	37,055	.375	.484	0.78	.74	.46
Neither	2,249	.042	.201	37,055	.042	.20	0.03	.06	.94
Catalan	2,249	.019	.136	37,055	.017	.128	0.21	.75	.45

C Descriptive statistics

Table C.1: Descriptive statistics

Variables	N	Mean	SD	Min	Max
Continuous Variables					
**Age**	37,055	48.61	15.12	18	88
**Personality (Big Five)**					
Extroversion	35,609	-.03	.99	-1.93	2.60
Agreeableness	35,722	-.02	1.04	-2.16	3.88
Conscientiousness	35,692	-.01	1.01	-1.45	3.78
Neuroticism	35,152	-.03	1.01	-2.58	2.08
Openness	35,809	0.11	1.05	-1.50	3.65
**Psychological Motivations**					
Need for Affect (NFA)	37,055	-.19	1.01	-5.31	2.56
Need for Cognition (NFC)	33,784	-.24	1.05	-4.65	2.80
**Ideology**	37,055	.16	1.07	-1.72	3.92
Binary variables					
**Gender, female**	37,055	.47	.50	0	1
**Turnout in regional elections 2012**	37,055	.85	.36	0	1
**Support for the Catalan secession**	37,055	.62	.49	0	1
Categorical (in dummies)					
**Province of residence**					
Girona	37,055	.19	.39	0	1
Tarragona	37,055	.20	.40	0	1
Lleida	37,055	.20	.40	0	1
Barcelona (reference)	37,055	.41	.39	0	1
**Educational Attainment**					
University	37,055	.24	.43	0	1
High School	37,055	.49	.50	0	1
Secondary (reference)	37,055	.27	.44	0	1
**Subjective Social Class**					
Upper/Middle - Upper	37,055	0.08	0.28	0	1
Middle	37,055	.54	.50	0	1
Lower/ Lower-Middle (reference)	37,055	.38	.48	0	1
**Monthly Household Income**					
More than 5.000€	35,830	.07	.26	0	1
Between 4.001€and 5.000€	35,830	.09	.29	0	1
Between 3.001€and 4.000€	35,830	.19	.40	0	1
Between 2.001€and 3.000€	35,830	.32	.46	0	1
Between 1.001€and 2.000€	35,830	.27	.44	0	1
Until 1€(reference)	35,830	.05	.23	0	1
**Origin**					
Immigrant	37,055	.17	.38	0	1
2n Generation	37,055	.16	.37	0	1
2n Generation mixed	37,055	.17	.37	0	1
Native (reference)	37,055	.50	.50	0	1
**Mother Language**					
Spanish	37,055	.38	.48	0	1
Both	37,055	.04	.20	0	1
Neither	37,055	.02	.13	0	1
Catalan (reference)	37,055	.56	.50	0	1

D Need for Affect Scale

D.1 Item wordings

Table D.1: 10-Item Need for Affect Scale (English)

Item	Item wording	AP/AV
1	I feel that I need to experience strong emotions regularly.	AP
2	Emotions help people to get along in life.	AP
3	I think that it is important to explore my feelings.	AP
4	It is important for me to be in touch with my feelings.	AP
5	It is important for me to know how others are feeling.	AP
6	If I reflect on my past, I see that I tend to be afraid of feeling emotions.	AV
7	I find strong emotions overwhelming and therefore try to avoid them.	AV
8	I would prefer not to experience either the lows or highs of emotion.	AV
9	I do not know how to handle my emotions, so I avoid them.	AV
10	Emotions are dangerous, they tend to get me into situations that I would rather avoid.	AV

Note: AP=Approach; AV=Avoidance.

Table D.2: 10-Item Need for Affect Scale (Spanish)

Item	Item wording	AP/AV
1	Siento que necesito experimentar emociones fuertes regularmente.	AP
2	Las emociones ayudan a la gente a tirar hacia adelante en la vida.	AP
3	Creo que es importante explorar mis sentimientos.	AP
4	Para mi es importante ser consciente de mis sentimientos.	AP
5	Para mi es importante saber lo que los otros sienten.	AP
6	Tiendo a tener miedo a emocionarme.	AV
7	Encuentro que las emociones fuertes me superan y, por lo tanto, intento evitarlas.	AV
8	Preferiría no experimentar ni los mejores ni los peores momentos de las emociones.	AV
9	No tengo muy claro como gestionar mis emociones y, por lo tanto, las evito.	AV
10	Las emociones son peligrosas, tienden a llevarme a situaciones que preferiría evitar.	AV

Note: AP=Approach; AV=Avoidance.

Table D.3: 10-Item Need for Affect Scale (Catalan)

Item	Item wording	AP/AV
1	Sento que necessito experimentar emocions fortes regularment.	AP
2	Les emocions ajuden a la gent a tirar endavant a la vida.	AP
3	Crec que és important explorar els meus sentiments.	AP
4	Per a mi és important ser conscient dels meus sentiments.	AP
5	Per a mi és important saber el que els altres senten.	AP
6	Tendeixo a tenir por d'emocionar-me.	AV
7	Trobo que les emocions fortes em superen i, per tant, intento evitar-les.	AV
8	Preferiria no experimentar ni els millors ni els pitjors moments de les emocions.	AV
9	No tinc molt clar com gestionar les meves emocions i, per tant, les evito.	AV
10	Les emocions són perilloses, tendeixen a portar-me a situacions que preferiria evitar.	AV

Note: AP=Approach; AV=Avoidance.

D.2 Correlation matrix

Table D.4: Correlation Matrix: NFA Scale

It.	1	2	3	4	5	6	7	8	9	10
1	1									
2	0.494	1								
3	0.319	0.603	1							
4	0.253	0.541	0.803	1						
5	0.233	0.471	0.643	0.683	1					
6	-0.065	-0.058	-0.029	-0.052	-0.108	1				
7	0.117	0.027	-0.000	-0.046	-0.086	0.522	1			
8	0.097	0.067	0.076	0.021	-0.020	0.409	0.570	1		
9	0.026	0.049	0.098	0.068	0.005	0.466	0.549	0.552	1	
10	0.024	0.070	0.103	0.066	0.009	0.421	0.508	0.532	0.649	1

Note: Factor loadings >.40 are boldfaced. reverse coding is used on AV items (6–10).

D.3 Factor loadings

Table D.5: Factor Loadings: 10-Item Need for Affect Scale (Affect-Approaching and Affect-Avoiding)

**Item**	**Factor 1**	**Factor 2**	**Factor 3**
1	**0.46**	−0.15	**0.66**
2	**0.65**	−0.27	0.23
3	**0.81**	−0.33	−0.15
4	**0.78**	−0.38	−0.26
5	**0.62**	−0.37	−0.16
6	0.18	**0.59**	−0.07
7	0.31	**0.69**	0.12
8	0.35	**0.62**	0.05
9	0.39	**0.68**	−0.09
10	0.38	**0.64**	−0.08

Note: Factor loadings >.40 are boldfaced. reverse coding is used on AV items.

E Need for Cognition Scale

E.1 Item wordings

Table E.1: 18-Item Need for Cognition Scale (English)

Item	Item wording
1	I would prefer complex to simple problems.
2	I like to have the responsibility of handling a situation that requires a lot of thinking.
3	Thinking is not my idea of fun.*
4	I would rather do something that requires little thought than something that is sure to challenge my thinking abilities.*
5	I try to anticipate and avoid situations where there is likely chance I will have to think in depth about something.*
6	I find satisfaction in deliberating hard and for long hours.
7	I only think as hard as I have to.*
8	I prefer to think about small, daily projects to long-term ones.*
9	I like tasks that require little thought once I've learned them.*
10	The idea of relying on thought to make my way to the top appeals to me.
11	I really enjoy a task that involves coming up with new solutions to problems.
12	Learning new ways to think doesn't excite me very much.*
13	I prefer my life to be filled with puzzles that I must solve.
14	The notion of thinking abstractly is appealing to me.
15	I would prefer a task that is intellectual, difficult, and important to one that is somewhat important but does not require much thought.
16	I feel relief rather than satisfaction after completing a task that required a lot of mental effort.*
17	It's enough for me that something gets the job done; I don't care how or why it works.*
18	I usually end up deliberating about issues even when they do not affect me personal.

Note: * Reverse scoring is used on this item.

Table E.2: 18-Item Need for Cognition Scale (Spanish)

Item	Item wording
1	Prefiero problemas complejos que simples.
2	Me gusta tener la responsabilidad de encargarme de una situación que requiera mucho esfuerzo
3	Pensar no se corresponde con mi idea de diversión.*
4	Prefiero hacer algo que requiera pensar poco que no algo que ponga a prueba mis habilidades a la hora de razonar.*
5	Intento anticipar y evitar situaciones en las que probablemente tenga que pensar profundamente sobre algo.*
6	Prefiero pensar en proyectos pequeños y diarios que no en proyectos a largo plazo.
7	Encuentro satisfactorio deliberar de forma intensa y durante largas horas.*
8	Sólo pienso lo justo y necesario.*
9	Prefiero hacer tareas que requieran pensar poco una vez las he aprendido.*
10	La idea de confiar en la razón para llegar lejos la encuentro atractiva.
11	Disfruto las tareas que requieren pensar en nuevas soluciones a problemas.
12	Aprender nuevas formas de pensar no me apasiona demasiado.*
13	Prefiero una vida llena de misterios por resolver.
14	La noción de pensar de forma abstracta la encuentro atractiva.
15	Prefiero una tarea intelectual, difícil, e importante a una que sea un poco importante pero que no requiera pensar demasiado.
16	Siento más alivio que satisfacción después de completar una tarea que requiere un gran esfuerzo mental.*
17	Para mí es suficiente que una cosa haga su trabajo; no me importa ni el cómo ni el por qué.*
18	Normalmente acabo deliberando sobre temas incluso cuando éstos no me afectan personalmente.

Note: * Reverse scoring is used on this item.

Table E.3: 18-Item Need for Cognition Scale (Catalan)

Item	Item wording
1	Prefereixo problemes complexos que no pas simples.
2	M'agrada tenir la responsabilitat d'encarregar-me d'una situació que requereixi molt d'esforç.
3	Pensar no es correspon a la meva idea de diversió.*
4	Prefereixo fer alguna cosa que requereixi pensar poc que no pas alguna cosa que posi a prova les meves habilitats a l'hora de raonar.*
5	Intento anticipar i evitar situacions en qué probablement hagi de pensar profundament sobre alguna cosa.*
6	Prefereixo pensar sobre projectes petits i diaris que no pas en projectes a llarg termini.
7	Trobo satisfactori deliberar de forma intensa i durant llargues hores.*
8	Només penso el just i necessari.*
9	Prefereixo tasques que requereixi pensar poc un cop les he après.*
10	La idea de confiar en la raó per arribar més lluny la trobo atractiva.
11	Disfruto les tasques que requereixen pensar en noves solucions a problemes.
12	Aprendre noves maneres de pensar no m'apassiona gaire.*
13	Prefereixo una vida plena de misteris per resoldre.
14	La noció de pensar de forma abstracta la trobo atractiva.
15	Prefereixo una tasca que és intel.lectual, difícil, i important a una que és una mica important però que no requereix pensar gaire.
16	Sento alleujament més que no pas satisfacció després de completar una tasca que requereix un gran esform̧ental.*
17	Per mi és suficient que una cosa faci la feina; no m'importa ni com ni per qué funciona.*
18	Normalment acabo deliberant sobre temes fins i tot quan no m'afecten personalment.

Note: * Reverse scoring is used on this item.

E.2 Correlation matrix

Table E.4: Correlation Matrix: NFC Scale

It.	1	2	3	4	5	6	7	8	9	10	11	12	13	14	15	16	17	18
1	1																	
2	0.485	1																
3	0.114	0.125	1															
4	0.173	0.185	0.438	1														
5	0.103	0.084	0.324	0.409	1													
6	0.171	0.190	0.244	0.307	0.334	1												
7	0.220	0.235	0.157	0.167	0.124	0.131	1											
8	0.133	0.168	0.342	0.375	0.309	0.290	0.210	1										
9	0.198	0.223	0.312	0.396	0.329	0.306	0.145	0.465	1									
10	0.156	0.177	0.136	0.131	0.050	0.067	0.229	0.122	0.085	1								
11	0.327	0.358	0.209	0.263	0.145	0.170	0.289	0.220	0.252	0.422	1							
12	0.179	0.201	0.337	0.354	0.273	0.247	0.201	0.378	0.375	0.166	0.337	1						
13	0.315	0.247	0.125	0.161	0.130	0.148	0.253	0.120	0.146	0.136	0.309	0.205	1					
14	0.282	0.202	0.216	0.229	0.181	0.189	0.308	0.205	0.209	0.231	0.355	0.288	0.419	1				
15	0.268	0.258	0.144	0.177	0.098	0.136	0.237	0.143	0.176	0.216	0.332	0.180	0.271	0.361	1			
16	0.112	0.116	0.200	0.240	0.207	0.184	0.097	0.226	0.247	0.094	0.156	0.241	0.098	0.115	0.049	1		
17	0.139	0.145	0.223	0.262	0.206	0.189	0.101	0.290	0.304	0.102	0.215	0.308	0.118	0.144	0.092	0.273	1	
18	0.163	0.170	0.125	0.131	0.099	0.105	0.359	0.161	0.100	0.210	0.246	0.148	0.218	0.274	0.206	0.044	0.076	1

E.3 Factor Loadings

Table E.5: Factor Loadings: 18-Item Need for Cognition Scale

Item	Factor 1	Factor 2	Factor 3
1	**0.47**	0.34	−0.38
2	**0.47**	0.30	−0.39
3	**0.49**	−0.27	0.09
4	**0.58**	−0.30	0.02
5	**0.45**	−0.33	0.03
6	**0.44**	−0.19	−0.08
7	**0.43**	0.25	0.19
8	**0.54**	−0.30	0.03
9	**0.57**	−0.30	−0.12
10	0.35	0.23	0.16
11	**0.59**	0.27	0.05
12	**0.57**	−0.16	0.05
13	**0.43**	0.28	0.05
14	**0.54**	0.25	0.20
15	**0.42**	0.28	0.06
16	0.34	−0.20	−0.06
17	**0.41**	−0.20	−0.06
18	0.36	0.24	0.24

Note: Factor loadings >.40 are boldfaced.

F Personality traits measures

F.1 Item wordings

Table F.1: Big-Five Inventory (English)

Item	Item wording	Factor
1	...is reserved.*	E
2	..is generally trusting.	A
3	...tends to be lazy.*	C
4	...is relaxed, handles stress well.*	N
5	...has few artistic interests.*	O
6	...is outgoing, sociable.	E
7	...tends to find fault with others.*	A
8	...does a thorough job.	C
9	...gets nervous easily.	N
10	...has an active imagination.	O

Note: * Reverse scoring is used on this item. E = Extroversion, A = Agreeableness, C = Conscientiousness, N = Neuroticism, O = Openness. The question wording is: "How well do the following statements describe your personality? I see myself as someone who..."

Table F.2: Big-Five Inventory (Spanish)

Item	Item wording	Factor
1	Soy reservado/a.*	E
2	Generalmente confio en los demás .	A
3	Tiendo a ser vago/a .*	C
4	Soy relajado/a, controlo el estrés .*	N
5	Tengo pocos intereses artísticos .*	O
6	Soy extrovertido/a, sociable.	E
7	Tiendo a buscar errores en los demás.*	A
8	Suelo acabar lo que empiezo.	C
9	Me pongo nervioso/a con facilidad.	N
10	Tengo una imaginación activa.	O

Note: * Reverse scoring is used on this item. E = Extroversion, A = Agreeableness, C = Conscientiousness, N = Neuroticism, O = Openness. The question wording is: "En qué medida las siguientes afirmaciones describen su personalidad?"

Table F.3: Big-Five Inventory (Catalan)

Item	Item wording	Factor
1	Sóc reservat/da.*	E
2	Confio en els altres.	A
3	Tendeixo a ser mandrós/a.*	C
4	Sóc relaxat/da, controlo l'estrés.*	N
5	Tinc pocs interessos artístics.*	O
6	Sóc extrovertit/da, sociable.	E
7	Tendeixo a buscar errors en els altres.*	A
8	Acostumo a acabar el que començo.	C
9	Em poso nerviós/osa amb facilitat.	N
10	Tinc una imaginació activa.	O

Note: * Reverse scoring is used on this item. E = Extroversion, A = Agreeableness, C = Conscientiousness, N = Neuroticism, O = Openness. The question wording is: "En quina mesura les seguents afirmacions descriuen la seva personalitat?"

F.2 Correlations across items

**Personality factor**	**Inter-item correlation**
Extroversion	0.52
Openness	0.26
Conscientiousness	0.37
Agreeableness	0.15
Neuroticism	0.49

G Correlations across all variables and measures

Table G.1: Correlation matrix: demographics, personality, NFA and NFC scales

Variables	Age	Female	Ideology	NFA	NFC	A	E	C	N	O
Age	1									
Female	-0.116	1								
Ideology (L-R)	0.132	-0.160	1							
NFA	-0.233	0.169	-0.137	1						
NFC	-0.234	-0.049	-0.057	0.409	1					
Agreeableness	-0.082	-0.096	0.072	-0.140	-0.054	1				
Extroversion	0.031	-0.120	0.030	-0.230	-0.085	0.151	1			
Conscientiousness	0.214	0.118	0.073	0.103	0.106	-0.144	-0.150	1		
Neuroticism	0.013	-0.143	0.027	0.091	0.148	-0.204	-0.018	0.074	1	
Openness	-0.045	0.054	-0.110	0.264	0.313	-0.058	-0.170	0.091	0.038	1

H Full regression models

Table H.1: Full regression models for the association between NFA, NFC, and Desire for Catalan independence

	DV: Desire for Catalan Independence					
	(1)	(2)	(3)	(4)	(5)	(6)
NFA	0.24*** (0.07)	0.25*** (0.08)	0.20*** (0.08)	0.22*** (0.07)	0.22*** (0.07)	0.15* (0.08)
NFC	0.005 (0.08)	0.08 (0.08)	0.07 (0.09)	0.001 (0.08)	0.07 (0.08)	0.07 (0.08)
NFA×NFC				−0.07 (0.08)	−0.09 (0.08)	−0.16** (0.08)
Age		0.005 (0.02)	−0.02 (0.03)		0.01 (0.02)	−0.02 (0.03)
Age squared		0.00 (0.00)	0.00 (0.00)		0.00 (0.00)	0.00 (0.00)
Female		0.06 (0.13)	−0.19 (0.13)		0.06 (0.13)	−0.20 (0.13)
Girona		0.96*** (0.15)	0.52*** (0.18)		0.96*** (0.15)	0.52*** (0.18)
Lleida		0.63*** (0.21)	−0.01 (0.21)		0.64*** (0.21)	−0.003 (0.21)
Tarragona		0.24 (0.18)	−0.01 (0.17)		0.24 (0.18)	−0.02 (0.17)
Income - 2		−0.50 (0.34)	−0.72** (0.33)		−0.51 (0.34)	−0.72** (0.33)
Income - 3		−0.20 (0.32)	−0.45 (0.31)		−0.20 (0.32)	−0.43 (0.31)
Income - 4		−0.14 (0.32)	−0.42 (0.32)		−0.14 (0.32)	−0.42 (0.32)
Income - 5		−0.35 (0.35)	−0.63* (0.33)		−0.36 (0.35)	−0.64* (0.34)
Income - 6		−0.96*** (0.36)	−1.04*** (0.35)		−0.97*** (0.36)	−1.06*** (0.35)
Education - 2		0.21 (0.17)	0.22 (0.16)		0.20 (0.16)	0.19 (0.16)
Education - 3		−0.23 (0.16)	−0.25* (0.14)		−0.23 (0.16)	−0.26* (0.14)
Agreeableness		−0.11 (0.07)	−0.13** (0.06)		−0.11 (0.07)	−0.13** (0.06)
Extroversion		0.11 (0.07)	0.09 (0.06)		0.11 (0.07)	0.09 (0.06)
Conscientiousness		−0.08 (0.06)	−0.02 (0.06)		−0.08 (0.06)	−0.01 (0.06)
Neuroticism		−0.03 (0.06)	−0.02 (0.06)		−0.03 (0.06)	−0.03 (0.06)
Openness		−0.04 (0.07)	−0.01 (0.07)		−0.04 (0.07)	0.004 (0.07)
Ideology (L-R)			−0.37*** (0.06)			−0.37*** (0.06)
Turnout in 2012			1.28*** (0.20)			1.28*** (0.20)
Origin - Spanish			0.32 (0.21)			0.31 (0.21)
Origin - Mixed			0.26 (0.22)			0.25 (0.22)
Origin - Catalan			0.59*** (0.23)			0.59*** (0.23)
Mother language - Both			1.99*** (0.20)			2.00*** (0.20)
Mother language - Others			−0.11 (0.48)			−0.12 (0.48)
Mother language - Catalan			2.35*** (0.18)			2.35*** (0.18)
Observations	34,081	31,565	31,565	34,081	31,565	31,565

Note: *p<0.1; **p<0.05; ***p<0.01 . The constant is omitted from the regression output.
