# Supplementary material for: Need for affect, need for cognition, and the desire for independence
Source: PLoS One. 2023 Feb 9;18(2):e0280457. doi: 10.1371/journal.pone.0280457 (PMC9910755; doi:10.1371/journal.pone.0280457)

MS. NO. PONE-D-21-32282 ENTITLED "NEED FOR AFFECT, NEED FOR COGNITION, AND THE DESIRE FOR INDEPENDENCE.'

April 13, 2022

Dear Academic Editor, I-Ching Lee,

Thank you for the opportunity to resubmit this manuscript. I believe the manuscript has benefited from the reviewers' helpful and thoughtful comments as well as your guidance. I have revised the manuscript, taking seriously each individual point raised by the three reviewers. My comments and responses are shown below each point.

I hope you agree that the manuscript has improved through this process and I am looking forward to your response.

Sincerely,

The Author

Date: April 13, 2022.

MS. NO. PONE-D-21-32282

The Editor

Thank you for submitting your manuscript to PLOS ONE. After careful consideration, we feel that it has merit but does not fully meet PLOS ONE's publication criteria as it currently stands. Therefore, we invite you to submit a revised version of the manuscript that addresses the points raised during the review process. Please consider reviewers' comments carefully and revise accordingly. I summarize major points raised by the reviewers.

(1) The research needs to situate itself in the previous literature on the support of political independence in Catalonia or elsewhere (e.g., reviewer 1's points 2, 4, & 7; reviewer 2' point in linking NFA and NFC to the identity and instrumental models).
- Thanks for your guidance on these points. I have bolstered the paper with relevant literature. On the connection between the identity-instrumental models and NFA-NFC, I would like to clarify that my goal was not to directly connect these two. Instead, my goal was to highlight that scholars implicitly assume that all individual process information in the same way. Rather than forcing everyone to fit with a model, the debate between identity or instrumental models might miss the fact that some individuals have a distinct predisposition to process information in a different, yet overlapping, way. Hence, it is useful to evaluate psychological motivations for information-seeking. The two most important psychological motivations that have previously been found to be associated with political behavior are NFA and NFC, which justifies the focus on them in this paper.

(2) The author(s) need to more carefully lay out what has been controlled for (and how the controls were linked to the previous literature).
- The section "psychological and socio-political controls" further develops this.

(3) More detailed information should be given with regards to the development of the scales (reviewer 1' point 5, reviewer 2's comments regarding results that are not the foci of the authors' hypotheses).
- This is appropriately discussed in my response to the reviewers.

(4) I also notice that the two types of needs were highly correlated (r = .41) and feel that the interaction term is going to be problematic (how can you interpret something that is overlapping in nature that has interaction?) Also, judging from the interaction terms in the models, it is only in model 6 that this interaction becomes significant.
- I have implemented VIF for the main-effects model and the VIFs are 1.369 and 1.658 for the NFA and NFC coefficients, respectively. For the interaction models, the VIFs are 1.496, 1.635 and 1.245 for the NFA, NFC, and the NFA*NFC coefficients, respectively. Given that all of these inflation factors are below the rule of thumb of 10, I understand that correlation of variables and its interactive terms are not concerning with regards to the interpretation of the results. I have added this information in the foot of Table 1.

(5) I am also not quite sure that I understand the logic of your proposed hypotheses. If NFA is a "general motivation to approach or avoid emotions or emotional inducing situations and includes the beliefs that emotions are useful for shaping judgments and

behavior"; how does this general motivation translate to the positive attitudes toward independence? I am thinking that interaction terms of NFA and some demographic characteristics that may reflect the identity approach would be more coherent in the current framework. The same set of questions could be applied to the hypotheses of NFC. It seems to me that NFA and NFC catch different ways of processing information. However, how these processes could be linked to positive attitudes toward independence are not clearly laid out.

- My argument is twofold. First, support for secessionism involves an extreme position and a challenge to the *status quo*, which has been shown to be more likely among those with high NFA (see Maio and Esses, 2001: 586). Second, and more importantly, I believe that the associations might arise from the context of the 2012-2014 period in Catalonia. Looking at the Catalan context during the period 2012-2014, support for the Catalan self-determination movement offers individuals who favor the secession—relative to those who are indifferent or against it—numerous opportunities to engage in emotion-inducing activities. Pro-independence mobilization involved participating in major demonstrations, taking part in marches throughout the country, and organizing and participating in unofficial referendums, all of which are events that induce strong emotional-inducing activities among those who join them. A review of some examples to illustrate this point can be found in the manuscript.

  The unionist movement only began mobilizing against the independence in 2017 – before that time only a very small minority of unionists actually took part in any emotional-inducing activity. Even after 2017, pro-unionist activities have been marginal compared to the mass mobilizations among pro-independence groups (i.e., many of which came as a response of the Spanish judicial activities against secessionists).
  I have revised the text in the paper to clarify these points and added a footnote to clarify that the pro-independence mass mobilization contrasts with the small-magnitude activities among pro-unionists throughout the same period and, in particular, during the period under study here, in 2013.

- Finally, I would like to thank you again for your detailed guidance throughour the review process, and I hope you appreciate the improvements in this revision process. I am looking forward to your response.

Reviewer 1

(1) This study provides results suggesting that some particular psychological attributes ("need for affect" and "need for cognition") might contribute to the expressed support for political secession on a Western European democracy. It does so by studying the relationships between these individual psychological traits and the willing to secede on a huge sample (N¿33.000) of Catalonian citizens who answered an online survey, during several months at 2013, at the peak of the secessionist effervescence that erupted in that Northeastern Spanish region at that time.

The study is well written, wisely argued and the technical analysis of the findings appears professional and sound. The justification for introducing psychological vectors as possible additional mediators of political decisions in turmoil situations is substantiated using the available literature on the issue. The discussion of the consistency and implications of the main findings, as well as some of its limitations, is also cogently presented.

The findings deserve to be known because they suggest that behind an unexpected urge for secession, in addition to identity, economic or other political vectors, individual psychological traits related mainly to emotional appetites played a non trivial role on a particular moment, at least, of the lively and enthusiast Catalonian secessionist movement (during several years, till it did'nt success failing to attain its goals).

- Thanks for the comment!

(2) At the introduction and through the whole paper the desire for political independence in Catalonia is described as if it was an spontaneous and grass-rooted movement. It obviates that it had a continuous and firm direction by a Regional Government and an Autonomous Parliament both dominated by secessionist parties. As an example, Figure 1 is used to illustrate the abrupt increase of the willing to secede between 2010-2013, neglecting to specify that such abrupt increase was, in fact, at 2012-2013, around the regional elections of 2012 that were called by a nationalist-lead Government that had changed from a decades-long moderate politics centered on enhancing devolution from Spain to a direct secession pathway.

- I have added references to this interpretation throughout the manuscript. As you will see in my response to your next point, I have added this idea together with relevant references in the introduction. Further in the text, I have added the literal wording suggested by the reviewer to describe Figure 1. It now reads: "The abrupt increase of the willing to secede between 2010-2013 is largely connected to the regional elections of 2012 that were called by a nationalist-lead Government that had changed from a decades-long moderate politics centered on enhancing devolution from Spain to a direct secession pathway."

(3) In this respect, the paper fully obviates a rich literature that has showed the relevant role played by regional elites and particularly the demanding outbid between regional nationalist parties on igniting and feeding the movement (Barrio and RdzTeruel 2017; Barrio and Field, 2018; Rdz. Teruel and Barrio 2021); or the crucial propagandistic role played by local media under direct or indirect influence of a highly resourceful

Regional Government (Oller et al, 2019).

- I appreciate the suggestions from the reviewer. I have added this important point in the introduction and added this aspect of the literature right upfront in the introduction. I hope this helps readers to situate the paper.

(4) The huge online sample is a convenient one but it's not representative of Catalonian population. This is duly recognized but has to be addressed in a much more consistent way. Despite the cautions taken by weighing and adjusting scores to those of a corresponding CEO profile in one of its regular Barometers surveys, the final profile of the sample is biased and consists on a peculiar cluster of Catalonian citizenry: educated, middle and upper class, mature age males, pro-leftist, pro-secession individuals born inside Catalonia clearly predominated. So, the results might illustrate tendencies and propensities of an important fraction of Catalonian citizenry but not those from the majoritarian population at all. In addition to the peculiar profile provided by these socioeconomic variables, a single measure illustrates that: 62% in favor of secession is an oddity, both in the long series of CEO and CIS surveys, and in all electoral results in the last decade. So the relevance of that should be discussed much more thoroughly.

- I agree with the reviewer that the sample cannot be considered as a representative sample of the Catalan society. The subsection "Participants" and the footnote 4 describes how the recruitment took place and it is obvious that the survey did not use a probabilistic method to recruit participants. I have added text in the section "Survey representativeness" to make sure that this is fully acknowledged right at the outset.

  Again, I acknowledge that 62 is not the same as 58.5, but the substantive discrepancy is not massive. To understand the value in the CEO, we must remember that mid-2013 was a unique time where the pro-independence movements was at its peak (see Figure 1). With this said, we should also take into account that the CEO might have suffered from pro-independence bias during those years and this value might be higher than it was in the actual population. Yet, evaluating this claim is outside the scope of this project, so I just take the BOP as the ground truth for weighting.

  I have added text that explicitly refers to the deviations from the online sample and the CEO sample; namely: "In summary, the convenience sample significantly over-represents males (53% over 49%), supporters of Catalan independence (62% over 59%), highly-educated respondents (24% over 21%, with a college degree and above) and upper/middle and upper-class respondents (8.3% over 7.7%), and under-represents respondents of non-Catalan origin (18% over 21%), in comparison with the probabilistic sample" (p.14). In my opinion, the differences between the weighted convenience sample and the probabilistic survey are existent but not substantively large.

Despite the small magnitude of the discrepancies, I have added some text discussing the potential impact of these differences in p. 14 by clearly discussing the assumptions required for the validity of the regression coefficients.

(5) The author avoids contrasting the potencies of his main psychological variables here with other personality vectors previously studied by him in another study (Barceló 2017), on the same sample. That's particularly surprising because his main predictor there "Agreableness", is highly charged by emotional tones, and emotion appetites play a crucial role on his interpretation of the findings. In addition, the absence of a proper discussion of the role played by "Need for cognition" and its plausible relation to "Openess", Intelligence or Education levels is also disappointing, because it reduces the analyses of individual psychological factors to a very narrow and perhaps inconsistent display. So, a full treatment and discussion of these complementary measures seems peremptory and might perhaps add to the role proposed for individual psychological factors on political options.

- The lack of focus on the relationships between NFA and NFC, and personality traits is because I was not expecting a strong correlation among them. For instance, in the seminal paper by Maio and Esses (2001), the authors reported moderate-small correlations between the Big-5 and NFA. Further, they conclude that the Big 5 and the NFA could potentially be correlated but are not redundant.

  Using the survey data, we can see that a simultaneous regression analysis reveals that the personality dimensions together explained only a small portion of the variance in the need for affect: $R2 = .07, F(5, 32416) = 508, p < .001$; and in the need for cognition: $R2 = .13, F(5, 32416) = 953, p < .001$. I have added this information in a footnote to explicitly mention that the study of psychological needs and personality traits belong to different studies due to their lack of redundancy and their distinct conceptual and empirical background.

  An interesting point is that the personality traits and the NFA and NFC scores are all standardized in the regression models that can be found in the online Appendix H. The estimated coefficient of NFA was 0.24, and the interaction coefficient 0.16. Because coefficients are standardized, these can be directly compared to the effects of personality traits. As we can observe, the effect of NFA on support for pro-independence is about double the size of Agreeableness, which is the only personality trait that is significantly associated with support for independence. I have added text mentioning this at the end of the Results section.

  Additionally, education has been found to be predicted by NFC in earlier work, which is consistent with theoretical expectations. However, it has also been found that educational achievement and NFC, while correlated, are conceptually distinct constructs and not redundant (Hawthorne et al. 2021). Consequently, it is important that the regression models control for respondents' education. I have also added text discussing this point in the section "Psychological Motivations

and the Desire for Independence."

(6) No references are given about translation and validation of the scales used to measure "need for affect" and "need for cognition". The Catalan and Spanish translations (Supp. Information) are very poor, with obvious ortographical and gramatical errors, and that should not be obviated when discussing limitations and weaknesses of the findings.

- I have gone through the items once again, and identified two typos related to LaTeXtranscription that are present in the manuscript but were not present in the original survey. For instance, the item 16 reported the typo "esform̦ental" while in the survey it was correctly written "esforç mental".

  Note that items (as it happens with the original English-version scales) are brief and succinct to fit in the survey format. However, I cannot identify the ortographical and gramatical errors mentioned by the reviewer in the scales. If the reviewer could kindly indicate where these errors are, I would be happy to ascertain whether they are typos related to the manuscript or they were also present in the original survey and, if the latter, I would openly acknowledge them in the discussion section of the paper.

  With this said, the validity of the scales can be ascertained from the factor loadings in the Appendices D.3 and E.3. In the Appendix D.3, we can observe that the loadings follow the expected Emotional Approach and Emotional Avoidance subscales. Given that the patterns of items follow what we should expect based on previous literature, it seems that the Need for Affect Scale that was employed in the survey captured the same underlying concepts as the English scale.

  With regards to the Need for Cognition, the Appendix E.3 also shows the loading factors associated with each item. We can observe that most items load on the same factor, which indicates an agreement across all items. In the end, it seems that all 18 items contributed to the variance of the underlying concept of need for cognition.

  We should note that if respondents had not understood the meaning of the items, we would see no patterns in the factor loadings or, at least, we would see patterns that would not be consistent with our expectations. Given that this is not what we see, this suggests me that people could perfectly understand the items and respond accordingly. Note that this would be true even in the presence of some typos, ortographical, or grammar errors.

(7) References to previous studies that have explored differential emotional landscapes characterizing the secessionist and unionist segments of Catalonian citizenry seems mandatory at the discussion (Oller et al 2018, Tobeña 2016, 2017).

- References to these important scholarly contributions have been added to the Discussion section.

(8) There is also an insufficient reference to studies that have showed important variations both on national identities and socioeconomic measures through the whole period of Catalonian secessionist effervescence, in longitudinal studies (i.e. Oller et al, 2019; 2020; 2021). The diagnoses of a transversal study made on a very particular moment should be compared and complemented with systematic and already well known longitudinal landscapes.

- Beyond this, I have added a new paragraph discussing what had been found from these other studies, and how it might be connected with the findings of this manuscript. Given that the cross-sectional data presented here is ill-suited to pinpoint time of shift in attitudes, I hope that the reviewer understand that the points made in the text are only speculative. With this said, I do think that the finding presented in the paper is consistent with the idea that in the surge of the movement (around 2012-13, where the survey was taken), regional elites could have constructed "an emotionally-driven environment to push for independence, which led those who were more passionate and more eagerly embraced emotions to join the movement. This is consistent with our finding and, certainly, this process might have come at the same time as many Catalans changed their dual Spanish-Catalan identities to hold an exclusive Catalan identity", which is found in other works such as Oller et al. (2019, 2020). While this is plausible, I would like to reiterate that I lack the longitudinal data necessary to link these attitudinal and psychological shifts in time.

(9) Regarding that, the lack of contrast of the obtained results with results from another similar online massive survey taken, at a different and crucial time during the secession bid, is a major weakness insufficiently discussed.
- I have revised the text to follow this advice. More specifically, I have discussed the interplay between this paper and this other work in several instances throughout the manuscript. If the reviewer believes that my references are still insufficient, I would be happy to consider more specific advice or suggestions in this regard.

Only if all these insufficiencies and caveats are adequately solved the present paper should go to the press. The findings, though transient and only suggestive, are clearly interesting and they deserve to be known.

- Thank you very much for your guidance and useful suggestions. I also hope you agree that the manuscript has improved through this process, and I am looking forward to your response.

MS. NO. PONE-D-21-32282

Reviewer 2

(1) A review of the manuscript "Need for Affect, Need for Cognition, and the Desire for Independence".

This study sets out to examine the role of two psychological factors in explaining intention to vote for Catalan independence from Spain. The study has an impressive sample size of over 35,000 Catalan respondents who are asked a battery of relevant questions, including two scales with good psychometric properties.

My general assessment of the paper is that it has a lot of potential but needs to be developed further, both theoretically and statistically.

- I appreciate the comments of the reviewer and her/his guidance in revising the manuscript.

(2) The author discusses two main reasons that have been suggested in the literature for why people support independence movements, the identity model, and the instrumental model. Why then, did the author choose to measure NFA and NFC in this study? There is no attempt to link NFA and NFC to the identity and instrumental models. In addition, a deeper dive into previous research relevant to the potential role of NFA and NFC in support for independence movements would have strengthened the paper.

- Thanks for asking this question. I have revised the text on p. 8 to clarify this point. My criticism to the dichotomy between the identity and the instrumental model in processing information is that scholars often assume that all individuals process the information in the same way. In this paper, I offer a possible solution to this controversy by looking into the individual-level differences in psychological motivations in processing political information. In other words, the theorizing offered here suggests that those who are high in NFA may process information in a different way that those low in NFA, which might lead to a different political behavior – in this case, support for independence.

- I have focused on NFA and NFC because these have been found by earlier work to be highly relevant in shaping political behavior (see, for instance, Arceneaux and Vander Wielen, 2013), so they were plausible candidates to similarly influence pro-independence voting.

(3) The study includes the ten-item personality inventory without explaining why or what the results mean. Same can be said for some of the other demographic and attitudinal variables.

- I have added new text in the section "Psychological and socio-political controls." I have further developed what the personality traits mean, as well as I have further justified the inclusion of some socio-political controls.

(4) As for the reporting of data I would have liked to see descriptive statistics (not just correlations in the appendix) and reporting of explained variance in the regression tables.

- The descriptive statistics with full detail are provided in the online Appendix C.

- Reporting the explained variance is not straightforward because the models reported are logistic regression model. As the reviewer surely knows, R squared is a useful metric for linear regression, but does not have the same meaning in logistic regression. Statisticians have come up with a variety of analogues of R squared for multiple logistic regression that they refer to collectively as "pseudo R squared", yet there is no consensus on which one is the best and their usefulness. For this reason, I chose not to report them.

- With this said, I am happy to share the results with the reviewer and, if the reviewer feels strongly to include them in the paper, I would be happy to report them in the manuscript. The pseudo R2 for the main models are the following: llh = -6461.90208; llhNull = -7926.86193; G2 = 2929.91971; McFadden = 0.18481; r2ML = 0.08864; r2CU = 0.22451.

(5) The more detailed regression table in the appendix also reveals several curiosities I suggest the author incorporates formally into the paper. The role of left-wing ideology, higher education and income, turnout in 2012 (what does that stand for?), and the clear importance of speaking Catalan.

- The reviewer is right. I have added a paragraph at the end of the results section summarizing the main findings from the full regression model.

(6) I was quite puzzled by the high predicted support from the models. While 62% of the sample supported independence for almost all the predictions mentioned in the manuscript they are much higher than that (usually around 85%). Maybe there is a clear statistical explanation for this, but I think it has to be provided.

- The reviewer is right that the discrepancy between the predicted values in Figure 2 and the average value is an oddity that requires explanation. In fact, the oddity is created by the control values used to create the simulated data. As mentioned in the manuscript, "Figure 2 shows the change in the predicted value of support for the independence for an individual who takes the median value across the categorical or ordinal control variables and the mean in the continuous control variables. In practice, this is equivalent to a 48-year-old man who lives in the province of Barcelona, has an income at the third level of the scale (out of the 6 income levels), holds a university degree or higher, has an average score across the Big-5 personality traits, a left-winging ideological score, turned out to vote in the most recent elections, his parents and himself are born in Catalonia, and Catalan is his mother language."

  Because the median might be different from the mean when a variable is skewed, the predicted values might not reflect the average respondent but, in this case, a simulated respondent with a clear vote intention. Note that some of the most

significant predictors in the model such as birthplace, mother language, ideology, prior turnout, all end up defining a secessionist (Catalan speaker, born in Catalonia with both parents being also born in Catalonia, left-wing, and who turns out in a regional election).

To be clear, if I had chosen other values such as someone with a centrist ideology, who did not turn out in the last elections, had both languages as their mother tongue, and Catalan-born with one parent born outside of Catalonia, the slope in Figure 3 would be similar but the predicted values would start at at a lower point and end at a lower point. See this alternative figure below for the reviewer's consideration. If the reviewer strongly feels that replacing the graph is better, I would be happy to do. In any case, this does not affect the slopes, so the findings that the graph in the paper and this one report.

(7) Finally, I think it may be more appropriate to frame the main finding in terms of the only group that shows the weakest support for independence. The only group in the four-cell matrix of high-low NFC and high-low NFA is the "low-NFC and low-NFA" group.

- I have revised the text following this advice.
- Overall, I would like to thank you for your useful suggestions, and I also hope you agree that the manuscript has improved through this process. I am looking forward to your response.

 MS. NO. PONE-D-21-32282

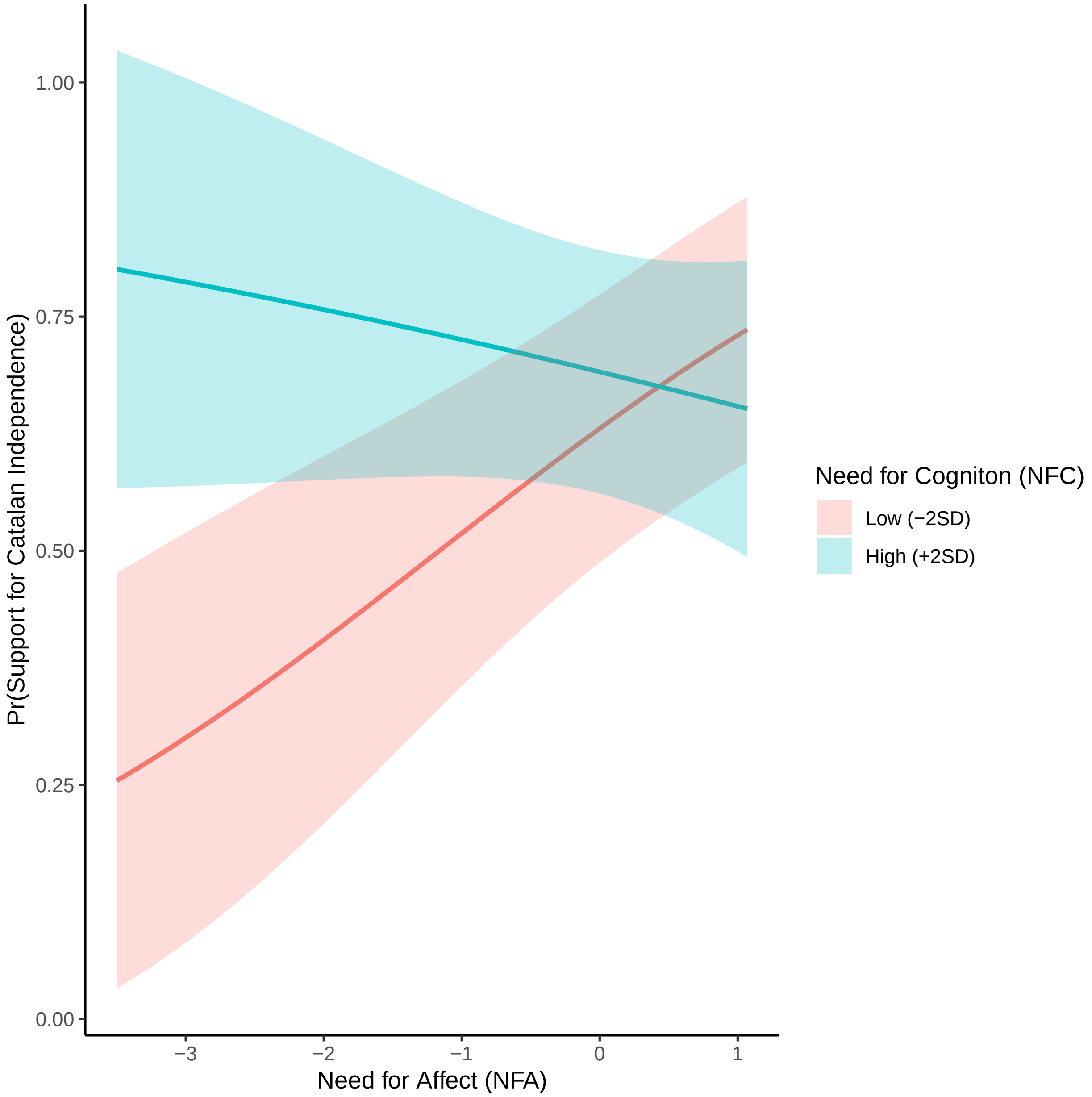

Supplement: Attachment — Submitted filename: NFA_on_Independence_response.pdf [file pone.0280457.s002.pdf]
