# Supplementary material for: Need for affect, need for cognition, and the desire for independence
Source: PLoS One. 2023 Feb 9;18(2):e0280457. doi: 10.1371/journal.pone.0280457 (PMC9910755; doi:10.1371/journal.pone.0280457)
Supplement: Attachment — Submitted filename: Memo.pdf [file pone.0280457.s003.pdf]

MS. NO. PONE-D-21-32282R1 ENTITLED "NEED FOR AFFECT, NEED FOR COGNITION, AND THE DESIRE FOR INDEPENDENCE.'

September 30, 2022

Dear Academic Editor, I-Ching Lee,

Thank you for the opportunity to resubmit this manuscript. I believe the manuscript has benefited from the reviewers' helpful and thoughtful comments as well as your guidance. I have revised the manuscript, taking seriously each individual point raised by the reviewers. My comments and responses are shown below each point.

I hope you agree that the manuscript has improved through this process and I am looking forward to your response.

Sincerely,

The Author

Date: September 30, 2022.

The Editor

Thank you for submitting your manuscript to PLOS ONE. After careful consideration, we feel that it has merit but does not fully meet PLOS ONE's publication criteria as it currently stands. Therefore, we invite you to submit a revised version of the manuscript that addresses the points raised during the review process.

The current manuscript, though, has improved, according to reviewer 1, is not evaluated favorably by reviewer 3. I read the manuscript myself and agreed with the major concerns raised by reviewer 3. Please explain carefully how psychology needs could be linked with support for the independence and let this explanation be the focus of your introduction.

- Thanks for your guidance on these points. I have added a more developed theoretical explanation in the theory section, as well as in the introduction. To be clear, the theoretical framework proposed here is that NFA and NFC are connected to the outcome of interest, support for the independence, conditional on the Catalan movement in 2013. In 2013, the movement was characterized by two major factors: the monopolization of the public space by secessionist symbols and the presence of large-scale pro-independence mobilizations. These conditions constitute the scope conditions of the paper and, as I argue, allowed NFA and NFC to be associated with pro-independence support. I hope the reviewer and the editor appreciate these clarifications in the paper.

It seems to me that the proposed associations of the need for affect and need for cognition do not pertain only to the case in Catalonia and if you could find additional data to support/replicate the current findings, it will greatly increase the merits of the current manuscript.

- I agree that the associations that are reported in this paper could travel well to other papers. In a way, the argument made in the paper is not a Catalan-specific argument but a general argument about behavioral tendencies in situation of an emergence of secessionist or transgressing movements. In my view, the generalizibility of the argument and, therefore, the expected generalizibility of the findings is a merit (and not a demerit) of the manuscript. With this said, I am not aware of any survey data that has included the NFA and the NFC in a similar context that could be used to test or replicate the same argument in a different context. If the editor or any of the reviewers are aware of this, I would be happy to add a new section. Otherwise, I have to leave this point for further research. The lack of comparable data highlights the novelty of the data analyzed in this paper. It is the first time, to the best of my knowledge, that the NFA and NFC scales are embedded in a political survey in the context of an emerging secessionist or transgressing movement. The advantage of this is that, for the first time, the associations between NFA and NFC and the support for these movements can be examined, yet it comes at the cost of not being able to find comparative data to empirically define the scope conditions of such associations. Overall, I hope that the editor and the reviewer appreciate the contribution of this manuscript to the literature.

 MS. NO. PONE-D-21-32282R1

Reviewer 1

(1) All comments and suggestions have been thoroughly ans satisfactorily addressed. The paper has greatly improved and must go swiftly to the print.
   - Thanks for your support.

(2) My only suggestion is for the Abstract: It should refer to the absence of relation between NFC and support for independence .

   Some minor errors remain in the Catalan-language versions of questionnaires at the Appendix: i.e. "complexos" (instead of "complexes"; "requereixi" (instead of "requerixi"); Catalan sentences do not demand a proposition particle before direct complements.; the translation looks rather "formal" and "bureocratic", distanced from common use of the language. But that cannot be changed.
   - Thanks for your guidance throughout the revision process. I have implemented these changes.

Reviewer 3

(1) This is an interesting work connecting psychological underpinnings (of NfA and NfC) with the Catalonian independence support. The author uses a large n, non-representative adult sample to test the association between psychological motives and support for the secessionist movement in Catalonia. As much as one could find a number of interesting aspects in this manuscript, the theoretical disconnect makes it hard to comprehend the real research goal and the potential inferences in this study. I thus cannot be supportive of its publication in its current condition. I further explain my comments below.

First, the primary gap in this work is the disconnect between the psychological drives (i.e., NfA and NfC) that tap individual differences in the sample and preferences on the Catalonian secession. Generally, work that uses NfC or NfA relate these factors to thought processes (as used in the original seminal studies) or to mid-level concepts (e.g., Big5, different types of thinking processes, compassion/empathy). These concepts cannot be treated as factors that one just wants to control for in a study. There has to be some kind of theoretical reasoning linking how NfC and NfA explain actual preferences on secession. That is the biggest aspect missing in the current manuscript.

Second, Cognition and Affect are two of the basic constructs of thought processes that capture how and why people think/evaluate/prefer the way they do. These constructs explain the mechanism of whether a person's thinking process is driven by cognitive assessments or affective evaluations. Similar concepts such as the Need to Evaluate or Uncertainty Avoidance explain the mechanism of thinking process, but not the behavioral outcome such as vote choice (or support for independence). The author repeatedly tries to connect emotions and cognition with the behavioral outcome in the findings section, but the evidence and discussions are quite weak.

Since I don't think that there is a decent theoretical foundation associating NfC and NFA with support for independence, I may recommend the following alternatives: One option to link NfC and NfA to the support for independence may go through the Big5. One of the Big5 dimensions may promote higher/lower support for the independence. There is preceding work on how Agreeableness, Conscientiousness, or other factors may relate to ideologies, voting behavior, strategic decisions, and freedoms. Another alternative could be through the level of political learning and political knowledge. Both of these factors relate to thinking processes that NfC and NfA are associated with. Politically knowledge could reveal some interesting results.

- I appreciate the thoughtful comments and suggestions of the reviewer. The reviewer is raising important concerns on whether psychological needs such as NfA and NfC may conceptually have a direct effect on preferences and behavior.

  The theoretical reasoning of this manuscript considers that Nfa and NfC can be predictors of preferences and behaviors conditional on individuals' stimuli, experiences, or the context. In other words, in a given context, how you process information (cognitive assessments or affective evaluations) affects what you think about an issue and shapes how you behave.

Imagine a scenario where people watches an emotional advertisement that seeks to persuade them to donate money for an NGO. All else equal, we could reasonably expect that those high in NfA would be more likely to donate after watching the ad that those who are low in NfA. In other words, given a given context, NfA can co-vary with preferences and behavior. As the reviewer points out, this is an assumption made in the manuscript.

Translating this to the content of this manuscript, pages 8-12 elaborate on how the Catalan context makes individuals' psychological drives to co-vary with preferences toward secessionism.

"During the surge of the pro-independence movement (2011-2014), individuals would perceive that supporting the secession—and not opposing it—would increase the expected likelihood of participating in emotion-inducing social and political activities." I note that "pro-independence mobilization involved participating in major demonstrations, taking part in marches throughout the country, and organizing and participating in unofficial referendums, all of which are events that induce strong emotional reactions among those who join them." "Therefore, I hypothesize that those who score high in NFA were more likely to support—rather than oppose—the Catalan independence at the time of the survey." (p.10).

The arguments provided in the manuscript emphasize the political context as a stimuli that would call for people high in NFA but not those low in NFA to support the independence. Obviously, if individuals would perceive that supporting the union would increase the expected likelihood of participating in emotion-inducing social and political activities, we would then expect NFA to be associated with the support for the union. I, hence, acknowledge that the hypothesis are derived from a general theory of behavior but specifically applied to the context of the Catalan conflict as it was in 2013.

This manuscript is not the first to explore the direct effects of NFA and/or NFC on preferences and behaviors. Most obviously, the seminal work by Maio and Esses (2001) already considers a direct effect of NFA on preferences. They state that "people who like to experience emotions may be more inclined to possess extreme opinions regarding controversial issues" (538). In other words, given a controversial issue, Maio and Esses (2001)'s prediction is that those high in NFA would hold the extreme preference. This is a statement predicting a direct connection between NFA and a preference.

Furthermore, extant research documents direct associations between NFA and numerous attitudinal and behavioral outcomes such as:

- Lins de Holanda Coelho et al. (2018) examine the effect of affective traits, including NFA, on the use of different types of drugs.
- Rosenbaum and Johnson (2016) study the role of psychological drivers on the enjoyment of unspoiled stories. The authors find that those low on

need for cognition hold a selective preference for spoiled stories, whereas individuals with a high need for affect enjoyed unspoiled stories more.
- Cramer et al. (2021) study how NFA and NFC may be clinically relevant for persons at escalated risk for suicide.
- Yildizeli Topcu (2021) look at the role of NFA on nurse's care-giving approach.
- Kang (2020) investigates the effects of NFA on attitudes toward online games.

Likewise the numerous studies on the direct role of NFA on attitudes and behaviors, the role of NFC has been similarly examined across a wide number of disciplines.

Regarding the direct role of NFC on attitudes and behaviors, Zerna, Strobel and Strobel (2021) is illustrative as they conducted a systematic review covering over 100 studies analyzing the direct effect of NFC on well-being. The outcomes in the studies, which are summarized in this review, include all kinds of preferences and behavioral intentions such as religiosity, college dropout, study performance, vaccine uptake, conspiracy beliefs, smoking, self-reported health, alcohol consumption, materialism, sleep deprivation, life satisfaction, decisional procrastination, study satisfaction, psychological wellbeing, cancer recurrence worry, academic engagement, self-efficacy, among many others. The meta-analysis is available at: `https://psyarxiv.com/p6gwh/`

Overall, this manuscript goes together with extant literature treating the NFA and NFC as factors – or underlying psychological motives - that shape how people process information in a particular context, which affects what people think about an issue and how they behave. I have revised the manuscript by adding a footnote (ft. 4, pg. 8) that makes explicit that the directional hypotheses are contingent to the context of the emergence of the Catalan secessionist movement, and referred to the extant literature that makes a direct connection between these psychological motivates and attitudes and behaviors.

(2) On additional issue with this manuscript is the generalizability of the results. Can we relate the findings to other secessionist movements across the globe? Given that NfC and NfA are quite consistent psychological constructs, would they explain similar preferences in Canada, Scotland, or China? Or, could we say anything about Brexit? The authors touches upon that but could not develop the idea as the theoretical foundations are quite weak. If supported properly, I think that would be a quite important inference.

- I appreciate the thoughtful suggestion of the reviewer. I have expanded the paragraph on p. 26 to capture this idea. However, I have to keep this in the realm of speculation as further research will be required to evaluate the extent to which the findings from Catalonia will generalize to other secessionist movements or to other exclusivity movements such as the Brexit. Given the scope conditions of the Catalan context described in p. 9-10, namely the role of the regional

elites in stirring up secessionist demands and the monopolization of the public space through emotion-inducing activities by the secessionist movement (protest, demonstrations), I can only speculate that it seems sensible that these contextual conditions are required to allow for individuals high in NFA, and also those high in NFA and low in NFC, to b willing to support secessionist or transgressing movements.

See the paragraph on p. 26: "Another aspect that requires further investigation has to do with the generalizability of these findings to other secessionist movements. The Catalan case has been often regarded as a prototypical case of a self-determination movement in a developed country—i.e., a peripheral region that is relatively advantageous in economic terms and a language/cultural singularity within the country. Given that NFA and NFC are quite consistent psychological constructs, the evidence presented here might generalize to most similar cases such as Flanders, Quebec, the Basque Country, and Scotland. Following the theoretical framework, I could speculate that the connection between NFA and NFC on secessionism is likely to require a context similar to that lived in Catalonia in 2013, which involved the monopolization of the public space by secessionist symbols and the presence of large-scale pro-independence mobilizations. Further research will also be required to examine whether contexts such as the Brexit movement could have shown a similar relationship between NFA and NFC and Brexit support. Outside of the developed world, however, many secessionist movements outside the developed world are strongly influenced by the dynamics of armed conflict. Hence, further research should assess whether macro-level processes (e.g., armed conflict) could wash away the effect of psychological factors on support for secessionist movements."

 MS. NO. PONE-D-21-32282R1

References

Cramer, Robert J, Laura H Gunn, Andréa R Kaniuka and Susan Rasmussen. 2021. "Making the case for a preferences in information processing model of suicide." *Journal of Social and Clinical Psychology* 40(4):359–380.

Kang, Naewon. 2020. "Effects of Online Game Users' Need for Affect and Social Norms on their Attitude and Flow Experience." *Journal of Korea Game Society* 20(4):21–32.

Lins de Holanda Coelho, Gabriel, Paul HP Hanel, Roosevelt Vilar, Renan P. Monteiro, Valdiney V Gouveia and Gregory R. Maio. 2018. "Need for affect and attitudes toward drugs: The mediating role of values." *Substance use & misuse* 53(13):2232–2239.

Maio, Gregory R and Victoria M Esses. 2001. "The need for affect: Individual differences in the motivation to approach or avoid emotions." *Journal of Personality* 69(4):583–614.

Rosenbaum, Judith E and Benjamin K Johnson. 2016. "Who's afraid of spoilers? Need for cognition, need for affect, and narrative selection and enjoyment." *Psychology of Popular Media Culture* 5(3):273.

Yildizeli Topcu, Sacide. 2021. "Surgical Nurses' need for affect and their caregiving approaches." *Journal of Public Health* 29:703–708.

Zerna, Josephine, Anja Strobel and Alexander Strobel. 2021. "The role of Need for Cognition in wellbeing–A review of associations and potential underlying mechanisms.".
