# Supplementary material for: Need for affect, need for cognition, and the desire for independence
Source: PLoS One. 2023 Feb 9;18(2):e0280457. doi: 10.1371/journal.pone.0280457 (PMC9910755; doi:10.1371/journal.pone.0280457)
Supplement: Attachment — Submitted filename: response_memo_plosone.pdf [file pone.0280457.s004.pdf]

MS. NO. PONE-D-21-32282R1 ENTITLED “NEED FOR AFFECT, NEED FOR COGNITION, AND THE DESIRE FOR INDEPENDENCE.’

December 14, 2022

Dear Academic Editor, I-Ching Lee,

Thank you for the opportunity to resubmit this manuscript. I have revised the manuscript, taking seriously the last few points raised by the editor and reviewer 3. My comments and responses are shown below each point.

I am looking forward to your response.

Sincerely,

The Author

Date: December 14, 2022.

The Editor

Thank you for submitting your manuscript to PLOS ONE. After careful consideration, we feel that it has merit but does not fully meet PLOS ONE's publication criteria as it currently stands. Therefore, we invite you to submit a revised version of the manuscript that addresses the points raised during the review process.

I have secured two reviewers' evaluations of the current manuscript and while one endorses the publication of the manuscript, one is more skeptical. The reviewer suggests a stronger link of NFC and NFA with political attitudes or judgements and recommends some papers that may be relevant for citations. Please consider incorporate them in the current manuscript. My reading is that it is very close to be accepted for publication but some revisions are needed. In addition to the literature the reviewer suggested, changes to some wording may be incorporated.

On page 1, you refer NFC and NFA as traits but motivations throughout the manuscript, please replace traits with motivations.

Because you have a survey data set which was collected one time, please be very careful when you use the causal terms. In addition, in the sample section, you started with 37,535 participants being recruited, but this is not true. Information provided elsewhere suggested that you recruited at least 47,148 individuals or 93,469 (survey link being clicked). Please revise to remove the inconsistent information and making sure that it is clear that the number of 37,535 participants were the ones that are further analyzed.

- Thank you very much for these suggestions. I have replaced traits with motivations in the front-end of the manuscript. I have also removed the reference to 47,148 individuals in the main text to avoid confusion. The correct number of participants were 37,535. The remaining participants were excluded from the analysis for the reasons outlined in the text. I hope this clarifies the matter.

Reviewer 3

(1) Reviewer 3: I enjoyed reading the revised version of the ms. Although we see minor changes in the written text, the response letter gives a bit more context and detail to reviewer comments. I must admit that I am still skeptical about the strength of the theoretical connection between NFANFC and the behavioral support for secessionism. Two important concerns come to mind:

First, the author claims that we may not have a preference about what those who score high in NFC would desire for Catalan independence. I disagree because one of the principal aspects of NFC is that people high in need for cognition tend to have persistent attitudes that are resistant to counterarguments (Haugtvedt and Petty, 1992). Besides, those who score high in NFC tend of have judgments that are more thoughtful, and have stronger attitudes making their behavior more predictable (Petty et al., 1995). With that, why could not we have clear predictions about high NFC people's preferences for independence? The theory could lead us make expectations prior to empirical insignificant effects.

- Thanks for these considerations. I have added new text in the section where I connect NFC with support for independence. With this said, I don't think that there is enough theoretical support so suggest that NFC will be connected with

support for secession before seeing the results.

On the other hand, the acquisition of information among people high in NFC is more elaborated and reasoned, persuasion takes place through the systematic processing rather than the heuristic processing (Haugtvedt and Petty, 1992). In this way, opinions, once acquired, become less malleable to changes in the external environment. Besides, those who score high in NFC tend of have judgments that are more thoughtful, including judgments about politics (Sohlberg, 2019), and have stronger attitudes making their behavior more predictable (Shoots-Reinhard et al., 2015; Petty, Haugtvedt and Smith, 1995).

In other words, people high in NFC may hold stronger, more elaborated, and resistant opinions but, at the same time, more nuanced and moderate opinions.

Because their opinions are stronger, one could think that they might be more in favor of independence. Yet, because their opinions are also more moderate, one might think that they are not. Ex ante, it is unclear what we would have expected.

Second, I think the author needs to be selective in the work associating NFANFC and the behavioral outcomes. Going back to the original works of Cacioppo and Petty in 1980s up until today, there must have been more than a few hundred publications using these two psych domains across an array of attitudinal and behavioral outcomes. Yet, the author lists (in the response letter) some that vaguely relate to political attitudes or behavior. I thus recommend referring to recently published work on these and related psych domains in the field with relevance to political attitudes and judgments.

- Thanks for this suggestion. As the reviewer will understand, I do not have the space to go over every study that has been written since the 1980s. Yet, I have added a few additional citations that relate NFC and NFA to political attitudes and behaviors, including the references that the reviewer explicitly mentioned. If the reviewer would like to suggest some more references, I will be happy to add them as well.

One minor issue: On page 26, in the revised section, “outside of the developed word” is repeated.

- I have revised this typo. Thanks for catching this.

 MS. NO. PONE-D-21-32282R1

Reviewer 4

This is an interesting piece that builds on research that incorporates NFA and NFC into political decision making. The large-n survey is also very impressive and allows the authors to estimate the two-way interaction of continuous variables well.

- Thanks for your support.

References

Haugtvedt, Curtis P and Richard E Petty. 1992. "Personality and persuasion: Need for cognition moderates the persistence and resistance of attitude changes." *Journal of Personality and Social psychology* 63(2):308.

Petty, Richard E, Curtis P Haugtvedt and Stephen M Smith. 1995. "Elaboration as a determinant of attitude strength: Creating attitudes that are persistent, resistant, and predictive of behavior." *Attitude strength: Antecedents and consequences* 4(93-130).

Shoots-Reinhard, Brittany L, Richard E Petty, Kenneth G DeMarree and Derek D Rucker. 2015. "Personality certainty and politics: Increasing the predictive utility of individual-difference inventories." *Political Psychology* 36(4):415–430.

Sohlberg, Jacob. 2019. "Elections are (not) exciting: Need for cognition and electoral behaviour." *Scandinavian Political Studies* 42(2):138–150.
